# Supplementary material for: Content-rich biological network constructed by mining PubMed abstracts
Source: BMC Bioinformatics. 2004 Oct 8;5:147. doi: 10.1186/1471-2105-5-147 (PMC528731; doi:10.1186/1471-2105-5-147)
Supplement: Additional File 2 — The original results of the above study (non-essential files are deleted to keep the file size under the limit set by BMC bioinformatics). [file 1471-2105-5-147-S2.bz2 › chilibotAdditionalFile2/dip05/599ID10025408E10978/html/FKBP1A_TGFBR1.html]

 


 **FKBP1A** and **TGFBR1** 
  
Found 3 abstracts in PubMed, retrieved 3.  
 

 What does Google say? 
 PDF only 
| .edu only 

---

**Interactive relationship** (e.g. stimulation, inhibition, etc)

**Inhibitory relationship**- FKBP12  [ **FKBP1A** ] , the 12 kDa FK506 binding protein, is a ubiquitous abundant protein that acts as a receptor for the immunosuppressant drug FK506, binds tightly to intracellular calcium release channels and to the transforming growth factor beta TGF beta type I receptor  [ **TGFBR1** ] .  Ref: 11226255 Proc Natl Acad Sci U S A, 2001
**Neutral relationship**- FKBP12  [ **FKBP1A** ]  binds to ligand free TGF beta type I receptor  [ **TGFBR1** ] , from which it is released upon a ligand induced, type II receptor mediated phosphorylation of the type I receptor.  Ref: 8756725 Cell, 1996

**Non-interactive relationship** (e.g. studied together, co-existance, homology, etc.)

- Rapamycin potentiation of TGF beta induced growth arrest could not be explained by increases in TGF beta receptor levels or rapamycin induced dissociation of FKBP12  [ **FKBP1A** ]  from the TGF beta type I receptor  [ **TGFBR1** ] .  Ref: 12417722 Mol Cell Biol, 2002
